# Engineered magnetosomes fused to functional molecule (protein A) provide a highly effective alternative to commercial immunomagnetic beads

**DOI:** 10.1186/s12951-019-0469-z

**Published:** 2019-03-06

**Authors:** Junjie Xu, Lingzi Liu, Jinxin He, Shijiao Ma, Shuli Li, Zhanhui Wang, Ting Xu, Wei Jiang, Ying Wen, Ying Li, Jiesheng Tian, Feng Li

**Affiliations:** 1 State Key Laboratory of Agrobiotechnology, College of Biological Sciences, China Agricultural University, Beijing, 100193 China; 2 Beijing Key Laboratory of Biodiversity and Organic Farming, College of Resources and Environmental Sciences, China Agricultural University, Beijing, 100193 China; 3 College of Life Science, Huaibei Normal University, Huaibei, 235000 China; 4 Beijing Advanced Innovation Center for Food Nutrition and Human Health, College of Veterinary Medicine, China Agricultural University, Beijing, 100193 China; 5 Beijing Advanced Innovation Center for Food Nutrition and Human Health, College of Biological Sciences, China Agricultural University, Beijing, 100193 China

**Keywords:** Bacterial magnetic nanoparticles, Protein A, Surface display technique, Gentamicin sulfate, *Vibrio parahaemolyticus*

## Abstract

**Background:**

Magnetosomes (also called bacterial magnetic nanoparticles; BMPs) are biomembrane-coated nanoparticles synthesized by magnetotactic bacteria (MTB). Engineered BMPs fused to protein A (termed ∆F-BMP-FA) bind antibodies (Abs) automatically, and thus provide a series of potential advantages. However, no report so far has systematically evaluated functional applicability of genetically engineered BMPs.

**Results:**

We evaluated properties of ∆F-BMP-FA, and developed/optimized culture methods for host strain *Magnetospirillum gryphiswaldense* ΔF-FA, ∆F-BMP-FA extraction conditions, conditions for Ab conjugation to ∆F-BMP-FA surface, and procedures for antigen detection using ∆F-BMP-FA/Ab complexes (termed BMP-A-Ab). Fed-batch culture for 36 h in a 42-L fermentor resulted in yields (dry weight) of 2.26 g/L for strain ΔF-FA and 62 mg/L for ∆F-BMP-FA. Optimal wash cycle number for ∆F-BMP-FA purification was seven, with magnetic separation following each ultrasonication step. Fusion of protein A to BMPs resulted in ordered arrangement of Abs on BMP surface. Linkage rate 962 μg Ab per mg ∆F-BMP-FA was achieved. BMP-A-Ab were tested for detection of pathogen (*Vibrio parahaemolyticus*; Vp) surface antigen and hapten (gentamicin sulfate). Maximal Vp capture rate for BMP-A-Ab was 90% (higher than rate for commercial immunomagnetic beads), and detection sensitivity was 5 CFU/mL. ∆F-BMP-FA also bound Abs from crude mouse ascites to form complex. Lowest gentamicin sulfate detection line for BMP-A-Ab was 0.01 ng/mL, 400-fold lower than that for double Ab sandwich ELISA, and gentamicin sulfate recovery rate for BMP-A-Ab was 93.2%.

**Conclusion:**

Our findings indicate that engineered BMPs such as ∆F-BMP-FA are inexpensive, eco-friendly alternatives to commercial immunomagnetic beads for detection or diagnostic immunoassays, and have high Ab-conjugation and antigen-adsorption capacity.

**Electronic supplementary material:**

The online version of this article (10.1186/s12951-019-0469-z) contains supplementary material, which is available to authorized users.

## Introduction

Research on nanomaterials and nanotechnology has expanded rapidly during the past two decades, because of the diverse applications of these materials in biomedical, agricultural, environmental, and physiochemical areas [1]. However, toxic properties of certain nanomaterials, and waste products generated during the manufacturing process, may have adverse environmental effects. Many recent studies have addressed the ecotoxicological impacts of nanoparticles, and their potential bioaccumulation in plants and microorganisms [2, 3]. Biological methods have been developed for synthesis of nanoparticles and microparticles. A variety of organisms and materials, including bacteria [4, 5], fungi [6, 7], yeast [8], plant extracts [9], and waste materials [10], have been utilized as eco-friendly precursors or processing tools for synthesis of nanoparticles with potential clinical or industrial applications. Most of these biological methods involve precipitation in specific matrices, although some are based on simple chemical reactions [11]. When whole cells are used, materials are typically formed outside the plasma cell membrane [12].

Magnetotactic bacteria (MTB) are a polyphyletic group of microorganisms that synthesize single-domain magnetite (Fe_3_O_4_) nanocrystals organized into magnetic organelles termed magnetosomes (or bacterial magnetic nanoparticles; BMPs) [13]. BMPs are coated by a biomembrane, and the nanoparticles are organic/inorganic hybrids whose formation is strictly controlled for uniform shape and size. In theory, MTB or their recombinant derivatives could be used to synthesize a series of composite nanoparticles with customized size, shape, composition, and functional groups, solely through fermentation processes [14].

Numerous chemical and genetic methods had been developed for modification of BMPs with enzymes, antibodies (Abs), receptor proteins, functional peptides, nucleic acid fragments, organic molecules, or beads [15, 16]. There has been increasing research focus on production of genetically engineered BMPs, using a method sometimes termed “magnetosome surface display technique”. This approach avoids the cross-linking processes that occur in chemical modifications, and maintains activity and orientation of functional molecules [17]. However, studies to date have not systematically evaluated functional applicability of genetically engineered BMPs, and there is some doubt regarding practical feasibility of magnetosome surface display technique.

We previously constructed a recombinant MTB strain, *Magnetospirillum gryphiswaldense* ΔF-FA, capable of forming an engineered BMP (here termed ∆F-BMP-FA) with protein A (termed Spa, because it is encoded by the gene *spa*) on its surface [18]. ∆F-BMP-FA spontaneously binds to most mammalian Abs containing Spa. In the present study, we investigated the practical applicability of ∆F-BMP-FA (as a representative engineered BMP) in immunomagnetic assay. ΔF-FA culture methods and ∆F-BMP-FA extraction conditions were developed and optimized. Ab conjugation abilities and detection limits of ∆F-BMP-FA BMPs were assessed and compared with those of commercial immunomagnetic beads.

## Materials and methods

### Bacterial strain and fermentation

Recombinant strain *M. gryphiswaldense* ∆F-FA [18] was cultured in a 42-L fermentor (BioFlo 110; New Brunswick Scientific, NJ, USA). Inoculum was cultured in sodium lactate medium as described previously [19]. Three sequential transfers with 10% (v/v) inoculation were performed, and inoculum was transferred to a 42-L fermentor. Optimized fermentation medium and feeding media, as determined previously [20]. Fermentation was performed with working volume 30 L, 10% (v/v) inoculation at 30 °C/100 rpm, and initial airflow 0.5 L/min. Once dissolved oxygen (dO_2_) decreased to 15%, airflow was increased to 1 L/min, and dO_2_ was subsequently maintained between 0 and 1% by regular agitation (added 20 rpm) every 2 h. pH was maintained at 7.0 by automated supplementation of feeding medium. After 12 h, 1 M of 7.5 mL isopropyl β-d-1-thiogalactopyranoside was added to induce the gene *mamF*-*proteinA* expression. OD_565_ (for estimation of cell density) and magnetic response (Cmag) were measured at 4-h intervals until termination of culture. Cmag was calculated based on measurement of maximum and minimum scattering intensities [21]. ∆F-BMP-FA yield was calculated as described previously [22].

### Purification, detection, and storage of engineered ∆F-BMP-FA BMP

Harvested cells were suspended in PBS (10 mmol/L; pH 7.4; 10 mL per g bacterial pellet). Cells were disrupted by ultrasonication (Ningbo Scientz Biotechnology Co.) (150 W; 99 runs; operation time 3 s; 5-s intervals between operations). ∆F-BMP-FA BMPs (hereafter referred to simply as “∆F-BMP-FA”) were captured from disrupted cell solution using a magnetic rack (Tianjin Beisile Chromatography Technology Development Center; Tianjin, China) (Fig. 2a). Solution was kept on the rack at 4 °C overnight, supernatant was removed, and precipitate was resuspended in PBS (100 μL/1 mg BMP), ultrasonicated (80 W; 50 runs; operation time 3 s; 5-s intervals between operations), and subjected to several rounds of magnetic capture/washing. At each round, protein concentration in supernatant was measured using BCA Protein Assay kit (Pierce Biotechnology/Thermo Fisher), until no further decrease was observed. Purified ∆F-BMP-FA were washed twice with distilled water under ultrasonication, captured with magnetic rack, suspended in 25% glycerinum, and stored at 4 °C.

Quantities of Spa present on ∆F-BMP-FA were estimated by one-step enzyme-linked immunosorbent assay (ELISA). A Spa standard curve was constructed (Additional file 1: Figure S1). 96-well microtiter plates (Nunc; Roskilde, Denmark) were incubated with successive dilutions (with PBS) of Spa standard solution (1, 0.5, 0.25, 0.125, 0.062, 0.031 µg/mL) at 4 °C overnight, washed 3× with PBST buffer (PBS containing 0.5% Tween-20), blocked with 250 μL gelatin for 1 h at room temperature, washed 3× with PBST buffer, then added with HRP-labeled goat anti-mouse IgG (100 µL; diluted 1:20,000 with PBS), incubated for 1 h at room temperature, and washed 5× with PBST. Color was developed using 100 µL TMB for 10 min at room temperature, and reaction stopped by adding 50 µL of 2 M H_2_SO_4_. Absorbance at wavelength 450 nm was measured on microplate reader (blank control: no Spa incubation). A two-parameter standard curve was constructed (Additional file 1: Figure S1). ∆F-BMP-FA were incubated with 1% BSA for 2 h at room temperature, in order to reduce non-specificity adsorption of Ab to ∆F-BMP-FA [27], washed 3× with PBST buffer, and 100 µL of ∆F-BMP-FA (10 µg/mL) was then added to plates for detection the amount of Spa based on the standard curve.

### Observation of engineered ∆F-BMP-FA by transmission electron microscopy (TEM)

A small amount of ∆F-BMP-FA was suspended in 1 mL deionized water and thoroughly dispersed by ultrasonication for 10 min. Ten µL of this suspension was dropped onto a copper mesh, left for 10 min, air-dried, and ∆F-BMP-FA were observed by TEM (model JEM-1230, JEOL; Tokyo, Japan).

### Hydrated radii and zeta potential of ∆F-BMP-FA

∆F-BMP-FA were resuspended in deionized water at concentration 0.01 mg/mL and thoroughly dispersed by 10 min ultrasonication. Hydrated radii and zeta potential were measured by Zeta-PALS (Brookhaven Instruments Corp.; Long Island, NY, USA).

### Conjugation of Abs to ∆F-BMP-FA, and detection

∆F-BMP-FA were suspended in 1 mL 1% BSA carbonate buffer solution, and incubated at 4 °C overnight, and captured with magnetic rack. Then, the ∆F-BMP-FA was resuspended in PBS, ultrasonicated 3×, and captured again. Suspension was added with 1 mL Abs (1 mg/mL). ∆F-BMP-FA were dispersed by ultrasonication, incubated with shaking (200 rpm) for 2 h at 37 °C, and captured with magnetic rack until BMP/Ab complex (hereafter termed BMP-A-Ab) was fully enriched. Ab concentrations in supernatants before and after conjugation were determined with BCA kit.

Coupling efficiency was calculated as μg Ab/mg ∆F-BMP-FA = (C_1_ − C_2_) × V/M, where C_1_ = Ab concentration before conjugation, C_2_ = Ab concentration after conjugation, V = reaction system volume, and M = magnetic body mass. C_1_ and C_2_ were calculated from standard curve y = kx. Standard curve was constructed using BSA standard solution included in BCA kit, with y = OD_562_, and x = corresponding protein concentration.

Amount of Ab coupling to ∆F-BMP-FA was also determined by ELISA. A standard curve for mouse anti-gentamicin Ab was constructed (Additional file 1: Figure S2). A 96-well microtiter plate was coated with 100 µL of successive dilutions (with 1% BSA carbonate buffer solution) of mouse anti-gentamicin Ab standard solution (192, 96, 48, 12, 6, 3, 1.5, 0.75 µg/mL), incubated at 4 °C overnight, washed 3× with PBST, blocked with 250 μL gelatin for 1 h at room temperature, washed 3× with PBST, added with 100 µL HRP-labeled goat anti-mouse IgG (diluted 1:20,000 with PBS), incubated for 1 h at room temperature, and washed 5× with PBST. Color was developed using 100 µL TMB for 10 min at room temperature, and reaction stopped by adding 50 µL of 2 M H_2_SO_4_. Absorbance at wavelength 450 nm was measured on microplate reader (blank control: no Ab incubation). A two-parameter standard curve was constructed (Additional file 1: Figure S2). 0.1 mg BMP-A-Ab was suspended in 1 mL containing 1% BSA carbonate buffer, then plate was added with 100 µL of this suspension (0.1 mg/mL) and incubated at 4 °C overnight. Amount of Ab coupling to ∆F-BMP-FA was determined based on the standard curve. Linkage rate was compared with that of commercial immunomagnetic beads fused with Spa (termed MB-A). Controls were ∆F-BMP-FA and MB-A not coupled with Ab.

Optimal conjugation conditions were determined based on testing and comparison of various buffers, temperatures, durations, and ∆F-BMP-FA/Ab mass ratios.

### Detection of *Vibrio parahaemolyticus* using engineered BMP/Ab complex

∆F-BMP-FA coupled with mouse anti-*Vibrio parahaemolyticus* Ab was used for detection of *V. parahaemolyticus* (ATCC 33847) (Vp), and detection rate was compared with that of MB-A. ∆F-BMP-FA and MB-A (each 1 mg) were suspended in Tris–HCl (pH 7.4) containing 0.1% Tween 20, added with 200 µL anti-Vp (O3 antigen) crude mouse ascites, dispersed by ultrasonication, and incubated with shaking (200 rpm) for 2 h at 37 °C to generate BMP/Ab complex (BMP-A-Ab) and magnetic bead/Ab complex (MB-A-Ab). Each complex was suspended in PBS and ultrasonicated 2× to remove non-specifically adsorbed Ab. Overnight culture of Vp was adjusted to initial OD_600_ = 0.5 and diluted to sequential concentrations 10^−6^ (~ 630 ± 25 bacteria), 10^−7^ (~ 47 ± 5 bacteria), and 10^−8^ (~ 5±0.5 bacteria). One mL of each diluted solution was spread on soft agar plate, and obtained colonies were counted. One mL of each diluted solution was mixed with BMP-A-Ab and MB-A-Ab, dispersed by ultrasonication, and incubated with shaking (200 rpm) for 30 min at 37 °C. Complexes were captured with magnetic rack and ultrasonicated 3× to obtain engineered BMP-A-Ab-Vp and MB-A-Ab-Vp. These complexes were resuspended in 200 μL PBS, spread on plates, and obtained colonies were counted. Detection rate (%) was calculated as N_s_/N_o_ × 100, where N_s_ = number of colonies corresponding to complexes spread on plate, and N_o_ = number of colonies corresponding to bacterial dilution spread on plate.

### Recovery of gentamicin sulfate by BMP/Ab complex

Coupling of ∆F-BMP-FA with anti-gentamicin Ab was performed based on optimal conjugation conditions. One mg ∆F-BMP-FA was added to 300 µL (1 mg/mL) mouse anti-gentamicin Ab (from mice immunized with bovine thyroglobulin-conjugated gentamicin; Bio-Rad Laboratories; Hercules, CA, USA) and 8 mL Tris–HCl (10 mM; pH 7.4). The reaction mixture was ultrasonicated (70-W cleaner) for 1 min for uniform dispersion, and incubated with shaking (200 rpm) for 2 h at 30 °C. A tube containing the mixture was placed over a magnet to isolate BMP-A-Ab, which was then washed 2× with 1 mL PBS buffer (10 mM; pH 7.4) under ultrasonication, and stored at 4 °C.

Gentamicin sulfate was recovered by BMP-A-Ab, extracted with ethyl acetate, and detected by double Ab sandwich ELISA. One mg BMP-A-Ab was suspended in successive dilutions (each 50 mL) of gentamicin sulfate solution (0.05, 0.025, 0.01, 0.005 ng/mL; respectively 2.5, 1.25, 0.5, 0.25 ng gentamicin). Each concentration was analyzed in triplicate. Reaction mixture was ultrasonicated (70 W) for 2 min, incubated with shaking (200 rpm) for 1 h at 37 °C, and placed over a magnet to isolate BMP/Ab-gentamicin complex. Complex was then washed 2× with 1 mL PBS (10 mM; pH 7.4), nonspecific adsorbed gentamicin sulfate discarded, complex added with 500 µL ethyl acetate, ultrasonicated (70 W) for 1 min, submerged for 5 min to extract gentamicin sulfate, supernatant transferred to a 1.5-mL tube, and incubated at 70 °C to evaporate ethyl acetate. Extracted gentamicin sulfate was dissolved in 100 µL ddH_2_O.

Double Ab sandwich ELISA was performed to detect extracted gentamicin sulfate, using a standard curve for gentamicin. A 96-well microtiter plate was coated with rabbit anti-gentamicin Ab (from rabbits immunized with KLH-conjugated gentamicin; Biorbyt LLC; San Francisco, CA, USA) and incubated at 4 °C overnight. The Ab was diluted 1:500 with 1% BSA carbonate buffer (pH 9.6; 0.05 mol/L). The plate was washed 3× with PBST, blocked with 250 μL gelatin for 1 h at room temperature, washed 3× with PBST, added with successive dilutions (each 100 µL per well) of gentamicin standard solution (1000, 333.3, 111.1, 55.6, 37.0, 27.8, 12.3, 4.1, 1.37, 0.46 ng/mL), incubated for 1 h at room temperature, washed 3× with PBST, added with mouse anti-gentamicin Ab (from mice immunized with bovine thyroglobulin-conjugated gentamicin; Bio-Rad Laboratories; Hercules, CA, USA) (1:2000 diluted), incubated for 1 h at room temperature, washed 3× with PBST, added with 100 µL HRP-labeled goat anti-mouse IgG (1:20,000 diluted in PBS), incubated for 1 h at room temperature, and washed 5× with PBST. Color was developed using 100 µL TMB for 10 min at room temperature, and reaction stopped by adding 50 µL of 2 M H_2_SO_4_. Absorbance at wavelength 450 nm was measured on microplate reader (blank control: no rabbit anti-Gentamicin Ab incubation). Immunoassay data were calculated using ELISA Calc software program, and fitted using four-parameter logistic equation. Double Ab sandwich ELISA was also performed to determine concentration of extracted gentamicin sulfate; in this case, 100 µL gentamicin sulfate suspension rather than gentamicin standard solution was added to microtiter plate. Recovery of gentamicin sulfate was calculated based on the standard curve.

## Results

### Culture of *M. gryphiswaldense* ∆F-FA in 42-L fermentor

Large-scale cultivation of MTB strains is difficult in general [23], and recombinant strains are even more difficult to culture than wild-type (WT) strains; therefore, engineered BMPs are not readily available, and too expensive for commercial applications. We successfully cultured *M*. *gryphiswaldense* MSR-1 in a 42-L fermentor [20], but submerged culture of recombinant MTB is uncommon. The only reported yield of engineered BMP from a surface display system was 7.5 mg/L, from a study by T. Matsunaga’s group using recombinant *Magnetospirillum magneticum* strain AMB-1 harboring plasmid pEML [24]. Recombinant *M. gryphiswaldense* strain ΔF-FA, which we constructed previously from MSR-1, was used in the present study to evaluate the possibility of fed-batch fermentation of recombinant MTB. Among the BMP-associated proteins in MSR-1, MamF is the most stable, and its gene and related mutant strains are commonly used in BMP surface display systems [25]. In our 2014 study, ∆F-FA chromosomal *mamF* gene was deleted, and fusion gene *mamF*-*spa* was expressed in recombinant plasmid pBBR-mamF-spa [18].

**Fig. 1:**
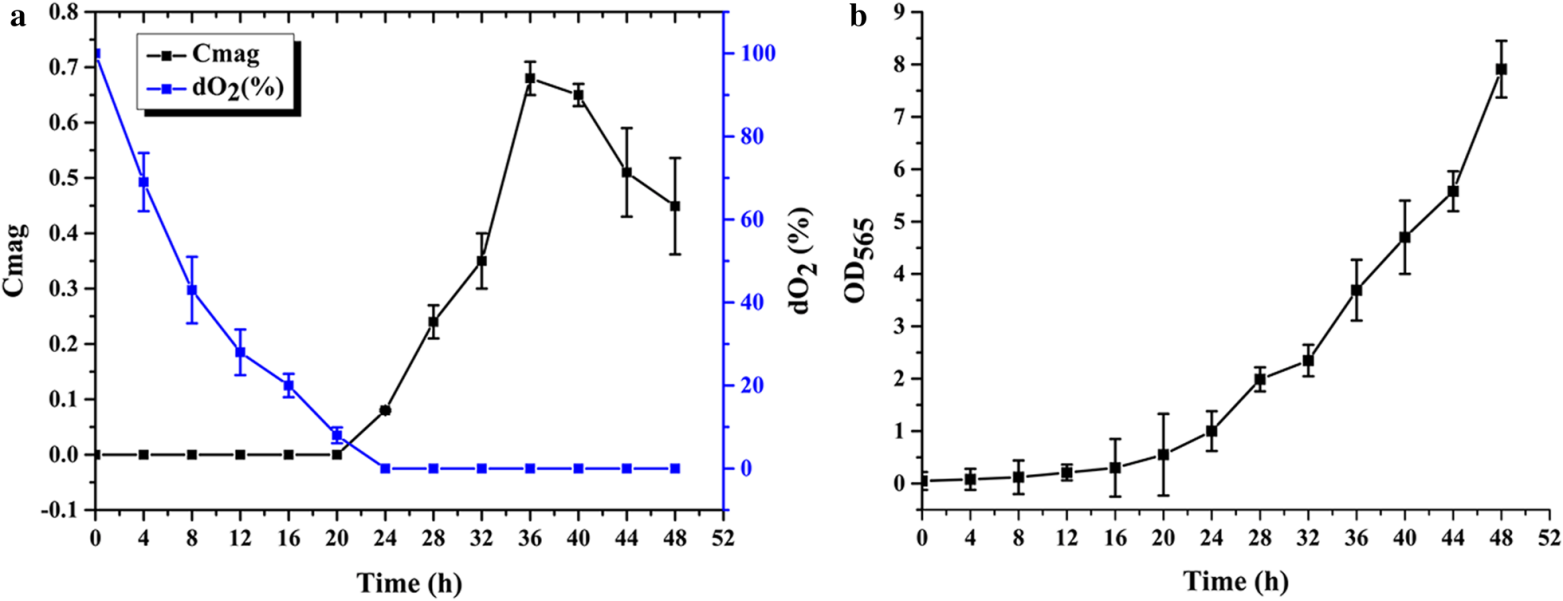
Submerged culture of *M. gryphiswaldense* ∆F-FA in 42-L fermentor: Cmag, dO_2_, and cell growth. **a** Cmag and dO_2_ values as a function of time. Cmag began rising above zero only after dO_2_ fell below 1%. **b** Growth curve. Cell density is estimated by OD_565_. Maximal Cmag value was 0.68. Maximal OD_565_ was 7.91. Total dry weight of collected cells was 67.8 g

We conducted fed-batch culture of ∆F-FA in a 42-L fermentor. Suitability of ∆F-FA for submerged culture was evaluated using a standardized strategy. Lactate was the only carbon source in MSR-1 fermentation medium. pH was maintained at 6.8 by automatic feeding of high-concentration lactate feeding medium. dO_2_ value was controlled between 0 and 1% by regulation of airflow and agitation speed (Fig. 1a). After 36 h culture, Cmag (parameter reflecting magnetic orientation) reached 0.68 and OD_565_ reached 7.91 (Fig. 1a, b). Low dO_2_ concentration significantly inhibited cell growth, similarly to WT MSR-1. Gradual increase of dO_2_ caused a significant increase of biomass. However, Cmag declined after reaching a peak value, whereas biomass continued to increase. Cell dry weight reached 2.26 g/L, and yield of engineered ∆F-BMP-FA was 62.29 mg/L. These findings indicate that ∆F-FA is potentially useful for industrial fermentation processes, although further optimization of culture conditions is necessary.

### Purification of engineered ∆F-BMP-FA

**Fig. 2:**
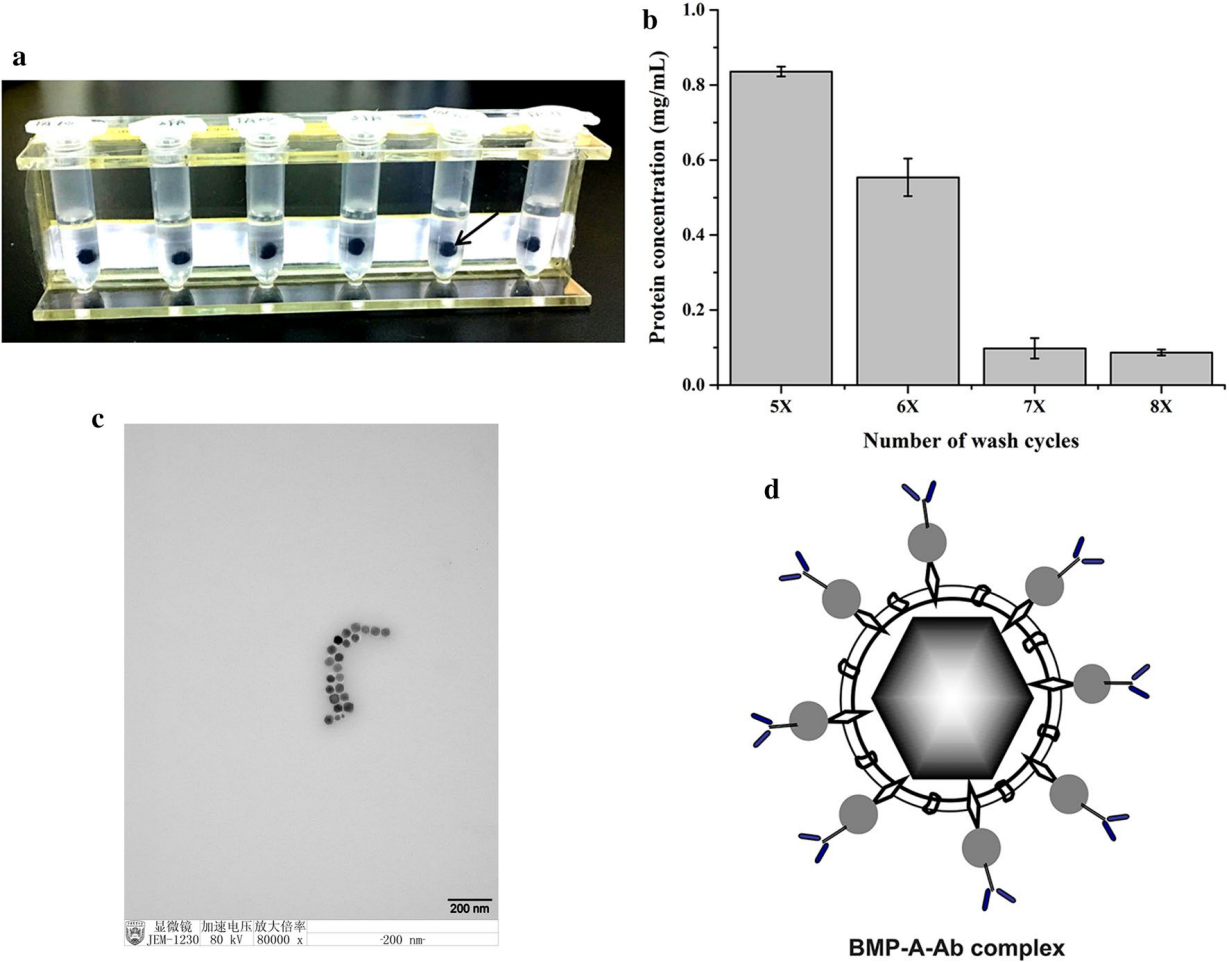
Purification of engineered magnetosomes (∆F-BMP-FA). **a** Magnetic rack containing purified ∆F-BMP-FA (arrow). **b** Protein concentration in supernatant following various numbers of wash cycles. Protein concentration fell below 0.1 following seven wash cycles, and no further protein was dropped as number of wash cycles increased. **c** Electron micrograph of purified ∆F-BMP-FA after seven wash cycles. Photo background reveals no stain; i.e., ∆F-BMP-FA was well purified. **d** Schematic diagram of BMP-A-Ab complex

Fusion proteins are always present on membranes of engineered BMPs. In this study, fusion protein MamF-Spa was present on ∆F-BMP-FA. In view of the ultrasonication steps involved in isolation and purification of ∆F-BMP-FA, we examined the possibility that the fusion protein or some part of it is dropped from BMPs during these steps, and affects the purification process. ΔF-FA cells were disrupted by higher-power ultrasonication, and ∆F-BMP-FA was then purified by several cycles of low-power ultrasonic bath, each followed by magnetic separation. As number of purification cycles increased, protein content in supernatant decreased. After seven purification cycles, supernatant protein content was nearly stable, indicating that no associated proteins were dropped from ∆F-BMP-FA (Fig. 2b). We previously obtained similar results for eight cycles of WT BMPs [22]. Thus, the fusion protein appears to be stable on ∆F-BMP-FA.

In a stable water-based suspension system, zeta potential (reflecting particle surface charge) of dispersed particles should be above + 30 mV or below − 30 mV. Surfaces of WT BMPs are negatively charged, and their zeta potential in water is approximately − 38 mV [18]. We examined polydispersity of ∆F-BMP-FA following each purification step. Zeta potential values of ∆F-BMP-FA were consistently near − 38 mV, and hydrated radius increased slightly as number of wash cycles increased (Table 1). In view of results for hydrated radii (Table 1), supernatant protein content (Fig. 2b), and TEM observation, the ∆F-BMP-FA was purification, no protein fragment on the background (Fig. 2c), seven wash cycles was considered optimal for purification process.

**Table 1:** Particle (hydrated radius) size, zeta potential, and polydispersity of ∆F-BMP-FA following various numbers of wash cycles

Number of wash cycles	Hydrated radius	Zeta potential	Polydispersity
5	674 ± 1.2	− 38.41 ± 0.05	0.279
6	783.7 ± 5.8	− 38.38 ± 0.56	0.307
7	789.1 ± 2.6	− 38.36 ± 0.43	0.282
8	820 ± 3.9	− 38.4 ± 0.32	0.261

One sample was prepared for each wash cycles and each sample was run 10 cycles (n = 10)

### Optimization of conditions for conjugation of Abs to engineered ∆F-BMP-FA

∆F-BMP-FA carry protein Spa on the surface and should automatically combine with Fc region of mammalian Abs and cause the Abs to be oriented with Fab region facing outward. As shown in a schematic diagram (Fig. 2d), this would make all Ab molecules more accessible to contact with target antigens [18]. However, only MamF protein fused with Spa and connected to Abs. In chemical modification methods, all surface molecules (including MamF) can be used to connect to Abs; therefore, ∆F-BMP-FA should bind a much smaller amount of Abs than do chemically modified BMPs. After optimizing binding conditions, we determined the maximal amount of Abs that ∆F-BMP-FA could bind. Reduced Ab proteins in supernatant, as detected by BCA kit, were considered to reflect the amount of Abs binding to ∆F-BMP-FA.

### Conjugation of Abs to ∆F-BMP-FA with varying purity

**Fig. 3:**
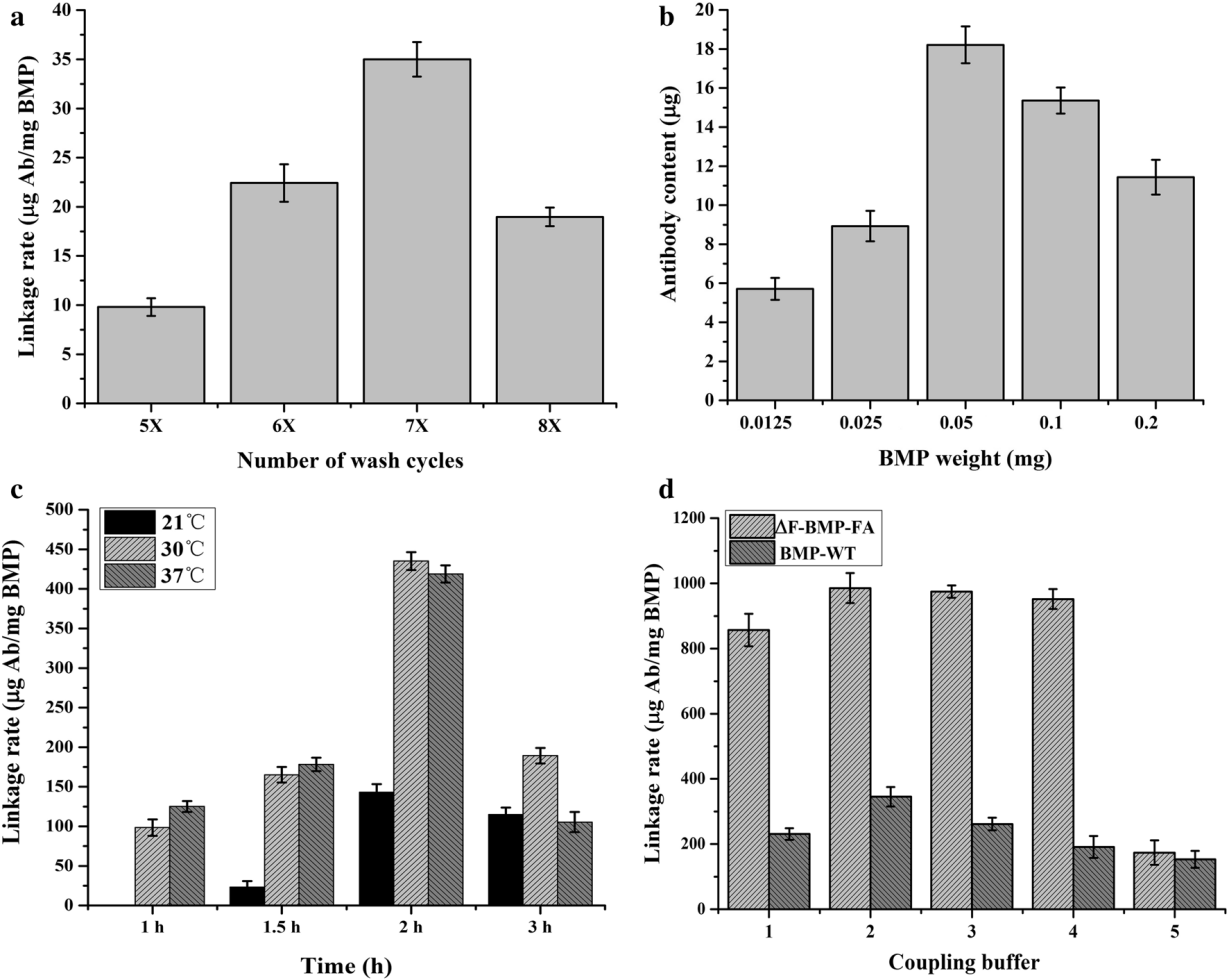
Optimization of conditions for conjugation of Ab to ∆F-BMP-FA. **a** Optimization of wash cycle number. Amount of linked Ab was maximal for ∆F-BMP-FA from seven wash cycles. **b** Optimization of ∆F-BMP-FA weight used for linkage to 300 µg Ab. Optimal weight ratio was 1:3. **c** Optimization of linkage temperature and time. Maximal linkage rate was observed for linking for 2 h at 30 °C. **d** Optimization of coupling buffer used for linkage of BMP to Ab. 1: 10 mmol/L PBS, pH 7.4. 2: 10 mmol/L Tris–HCl, pH 7.4. 3: 10 mmol/L HEPES, pH 7.4. 4: 10 mmol/L Na_2_HPO_3_-C_6_H_8_O_7_, pH 7.4. 5: 10 mmol/L KH_2_PO_3_-NaOH, pH 7.4. Buffer #2 (10 mmol/L Tris–HCl, pH 7.4) was selected for use in subsequent experiments

Rabbit IgG was coupled to ∆F-BMP-FA purified using various durations of ultrasonication, to determine maximal amount of Abs binding. ∆F-BMP-FA from various numbers of wash cycles were conjugated with IgG (Fig. 3a). ∆F-BMP-FA from seven wash cycles conjugated the highest amount of Abs, confirming that seven was the optimal number of wash cycles for purification process. This condition was adopted for all subsequent experiments. Presumably some other proteins on BMPs may interfere with binding of Ab to ∆F-BMP-FA at lower number of wash cycles, whereas excessive ultrasonication may impair ∆F-BMP-FA function at higher number of wash cycles.

### Optimization of amount of ∆F-BMP-FA

Various amounts of ∆F-BMP-FA (ranging from 0.0125 to 0.2 mg) were used to bind 300 μg Ab (a standard quantity used in many of our previous studies) in a 1-mL reaction system. Maximal Ab binding was obtained when 0.1 or 0.05 mg ∆F-BMP-FA was used (Fig. 3b). In subsequent experiments, 0.1 mg ∆F-BMP-FA was used because preparation of this amount involved less deviation. Interestingly, capacity of ∆F-BMP-FA to bind Ab was not correlated with Ab amount. One possible explanation is that ∆F-BMP-FA at higher concentrations gather together to form larger particles, with consequent reduction of specific surface area.

### Optimization of incubation time and temperature

Incubation times for ∆F-BMP-FA/rabbit IgG conjugation were compared at 21, 30, and 37 °C (Fig. 3c). Amounts of conjugated Ab were much higher at 30 °C and 37 °C than at 21 °C. Highest conjugation (linkage) rates were observed for incubation time 2 h. Optimal time/temperature conditions selected for ∆F-BMP-FA/Ab conjugation in subsequent experiments were 2 h at 30 °C.

### Optimization of coupling buffer

Several common buffers were tested and compared for use in ∆F-BMP-FA/Ab conjugation: 10 mol/L PBS (pH 7.4), 10 mmol/L disodium hydrogen phosphate-citric acid buffer (pH 7.4), 10 mmol/L 4-hydroxyethylpiperazine ethanesulfonic acid (pH 7.4), 10 mmol/L Tris–HCl (pH 7.4), and 10 mmol/L phosphate buffer (pH 7.4). In the buffer 10 mmol/L Tris–HCl (pH 7.4), 0.1 mg ∆F-BMP-FA was coupled to 98.55 μg Ab, as determined by BCA kit (Fig. 3d) (linkage rate of 985.5 µg Ab per mg ∆F-BMP-FA). This was the maximal amount of conjugated Ab per unit mass ∆F-BMP-FA under any tested condition. This buffer was therefore selected as optimal buffer for conjugation reaction.

For confirmation, amount of Ab linked to ∆F-BMP-FA was determined using standard curve of anti-gentamicin Ab from mouse (Additional file 1: Figure S2). Linkage rate determined in this way was 962.53 µg Ab per mg ∆F-BMP-FA, nearly the same as rate determined using BCA kit.

Binding of Ab to ∆F-BMP-FA was higher than in previous studies using chemical conjugation [26, 27]; suggesting that orientation of Abs on BMPs may compensate for smaller number of connecting groups.

### Amount of Spa on ∆F-BMP-FA

Spa standard curve was constructed based on one-step ELISA (Additional file 1: Figure S1). Spa content was estimated as 13.9 ± 0.03 μg/mg ∆F-BMP-FA, based on the standard curve. We further estimated, based on a recent report by T. Yoshino’s group [28], that one ∆F-BMP-FA contains > 130 Spa molecules. Each protein A molecule has five IgG-binding domains; therefore, one ∆F-BMP-FA could theoretically have > 650 Ab molecules, ensuring availability of large amounts of Ab for binding to engineered BMPs.

### Detection of *V. parahaemolyticus* using BMP/Ab complex

**Fig. 4:**
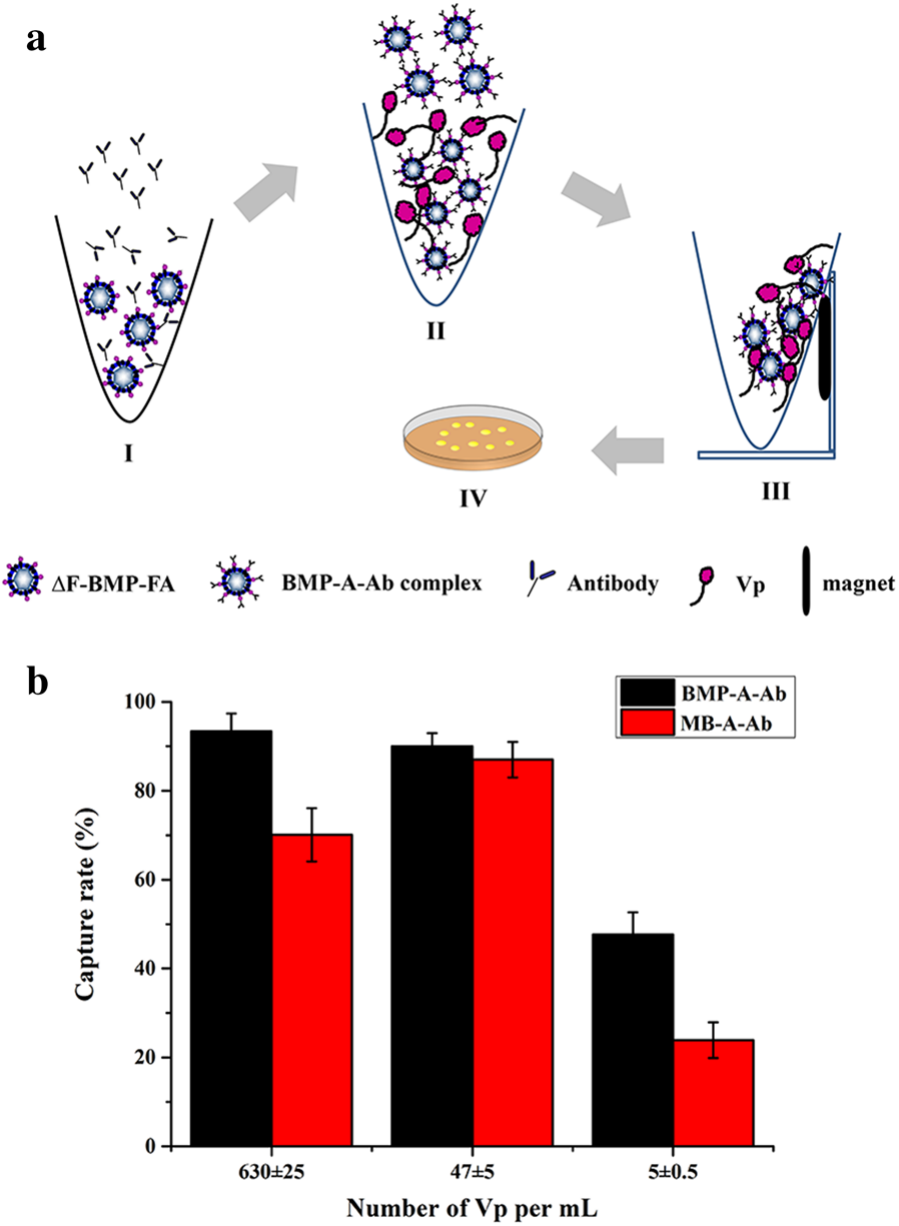
Detection of *V. parahaemolyticus* (Vp) by BMP-A-Ab in comparison with commercial immunomagnetic bead/Ab complex (MB-A-Ab). **a** Detection of Vp using ∆F-BMP-FA (schematic). Step I: ∆F-BMP-FA is coupled to anti-Vp Ab to construct BMP-A-Ab. Step II: BMP-A-Ab captures Vp. Step III: capture by magnetic rack. Step IV: captured Vp is resuspended and spread on plate. **b** Comparison of Vp capture rates for BMP-A-Ab vs. MB-A-Ab with three concentrations of Vp. Capture rate was consistently greater for BMP-A-Ab. Maximal capture rate was 93%, for Vp number 630 ± 25 per mL

Following optimization of linkage conditions for ∆F-BMP-FA/Ab complex (termed BMP-A-Ab), and achievement of high linkage rate, BMP-A-Ab was evaluated for pathogen detection. The pathogen *V. parahaemolyticus* (Vp) was selected as an experimental model for this purpose, using a detection process as illustrated in (Fig. 4a). Vp is a halophilic Gram-negative bacterium found in fish, crustaceans, and bivalve mollusks (particularly oysters), is often associated with food-borne diseases, and is a cause of severe food poisoning outbreaks worldwide. We compared the rate of Vp capture by BMP-A-Ab with that of commercial immunomagnetic beads fused with Spa (MB-A) (see recovery of gentamicin sulfate by BMP/Ab complex in the “Materials and methods”). ∆F-BMP-FA and MB-A were sterilized with ^60^Co, and crude mouse anti-Vp ascites was filtration-sterilized and conjugated to ∆F-BMP-FA and MB-A. The resulting BMP-A-Ab and MB-A-Ab complexes were used to capture Vp at three concentrations, and colonies were counted before and after capture. For Vp concentration 630/mL, capture rate was much higher for BMP-A-Ab (93%) than for MB-A-Ab (70%) (Fig. 4b). Detection sensitivity was 5 CFU/mL for both BMP-A-Ab and MB-A-Ab.

### Recovery of gentamicin sulfate by BMP/Ab complex

**Fig. 5:**
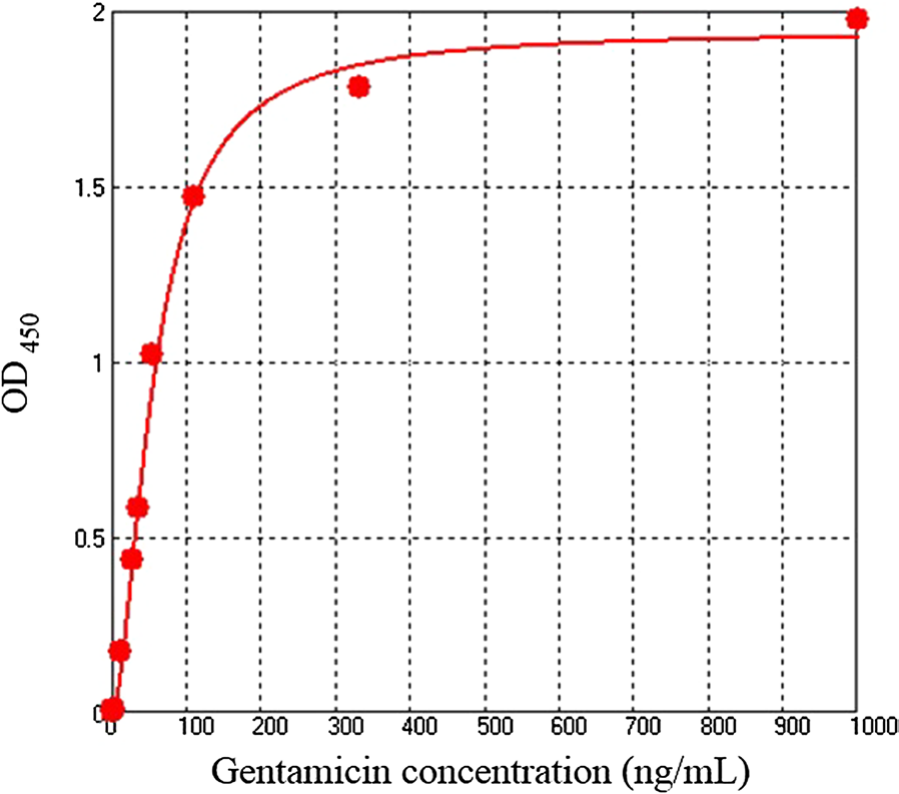
Gentamicin sulfate standard curve determined by double Ab sandwich ELISA

BMP-A-Ab also detected haptens, with gentamicin as example. Low-concentration gentamicin sulfate was enriched, ethyl acetate was used to extract gentamicin captured by BMP-A-Ab, and recovery rate was determined by double Ab sandwich ELISA. Gentamicin standard curve was constructed, and OD_450_ values from dilution gradient of gentamicin sulfate standard solution are shown in (Table 2). OD_450_ value for concentration 0.46 ng/mL (0.179) was close to that for blank control (0.178), and value for concentration 1.37 ng/mL (0.186) was close to that for 4.1 ng/mL (0.185). Lowest detection limit for gentamicin sulfate was therefore set as 4.1 ng/mL. A four-parameter gentamicin sulfate standard curve was constructed (Fig. 5). Equation of regression curve was y = (A-D)/[1 + (x/C)^B^] + D (A 1.9403, B − 1.67598, C 56.51, D − 0.00104, r^2^ = 0.99693, n = 3). Detection range was defined as 4.1–111.1 ng/mL. Recovery of gentamicin sulfate by BMP-A-Ab was calculated from the standard curve. Amounts of gentamicin sulfate extracted from 50 mL solution with various concentrations were: 0.664 ng from 0.05 ng/mL, 0.406 ng from 0.025 ng/mL, 0.466 ng from 0.01 ng/mL, and 0.00417 ng from 0.005 ng/mL (Table 3). Maximal recovery was 93.2% at gentamicin sulfate concentration 0.01 ng/mL. Gentamicin enrichment of BMP-A-Ab lowered the gentamicin sulfate detection line. The lowest detection line (0.01 ng/mL) was ~ 400-fold lower than that of double Ab sandwich ELISA.

**Table 2:** OD_450_ values from dilution gradient of gentamicin sulfate standard solution, determined by double Ab sandwich ELISA

Concentration (ng/mL)	1000	333.33	111.11	55.56	37.04	27.78	12.35	4.12	1.37	0.46
OD_450_	2.154	1.959	1.644	1.2	0.761	0.612	0.352	0.185	0.186	0.179
OD_450_ blank	0.178	0.178	0.178	0.178	0.178	0.178	0.178	0.178	0.178	0.178
OD_450_-OD_450_ blank	1.976	1.781	1.466	1.022	0.583	0.434	0.174	0.007	0.008	0.001
SD	0.024	0.064	0.14	0.057	0.099	0.093	0.030	0.011	0.008	0.006

**Table 3:** Recovery of gentamicin sulfate by BMP-A-Ab

Initial gentamicin sulfate (ng)	OD_450_ of extracted gentamicin sulfate (ng/mL)	Extracted gentamicin sulfate (ng)	Recovery rate of gentamicin sulfate (%)	RSD (%)
2.5	0.2295 (6.64)	0.664	26.56	2.8
1.25	0.2065 (4.66)	0.406	32.48	4.7
0.5	0.2005 (4.06)	0.466	93.2	8.3
0.25	0.1825 (0.00417)	0.0004	0.61	4.3

Three samples were prepared for each concentration level (n = 3)

Gentamicin sulfate recovery rate at concentration 0.01 ng/mL was compared for BMP-A-Ab vs. MB-A-Ab. Ab was replaced with mouse ascites containing anti-gentamicin Abs (prepared by Z. Wang’s group). ∆F-BMP-FA and MB-A are both able to directly use mouse ascites to construct magnetic particle/Ab complex (BMP-A-Ab, MB-A-Ab) without Ab purification process or concentration of antigen. Complexes were used for recovery of 0.01 ng/mL gentamicin sulfate. Recovery rates were 72% for BMP-A-Ab and 64.9% for MB-A-Ab; i.e., slightly higher for BMP-A-Ab than for commercial immunomagnetic beads.

## Discussion

Industrial fermentation involves adaptation of metabolic processes of microorganisms or cultured animal cells to generate products that are useful to humans. Fermentation products and processes, including biomass yield, extracellular metabolites, intracellular components, and substrate transformation, have applications in food industry and other industries [29]. Research and development of magnetotactic bacteria (MTB) over the past two decades have made possible synthesis of a new series of products (custom-made, highly uniform nanomaterials) based solely on fermentation processes [14]. MTB are typically difficult to isolate or culture because of their highly precise and restricted living conditions [23]. Results of the present study, however, suggest that both WT MTB [20] and recombinant MTB can be cultured on a large scale, thus facilitating practical applications of WT BMPs and engineered BMPs.

Considerable recent attention has been paid to BMP-based magnetic separation techniques and immunomagnetic assays [30]. BMPs are quite similar to commercial micro- or nano-sized immunomagnetic beads with core–shell structures, but generally display much stronger magnetism because their cores contain single-domain magnetic crystals and a much higher percentage of magnetite. BMPs, because of their strong magnetism and small size, are easy to manipulate and have high specific surface area [31, 32].

Another unique advantage of BMPs is that functional molecules can be expressed on their surface by genetic engineering methods. Three advantages of engineered BMPs over man-made magnetic particles and WT BMPs are: (i) expensive reagents are not required to link functional molecules to BMPs; (ii) engineering methods do not pose a risk of damage to functional molecules by reactions with chemical reagents; (iii) desired orientation of functional molecules is maintained. Although only parts of engineered BMPs are involved in linkage to functional molecules, the molecules show strong activity. For example, the amount of Ab binding to 1 mg ∆F-BMP-FA (engineered BMP) fused to Spa protein (foreign functional molecule) in the present study was very high (960 μg).

Because of the large amount of Ab bound to ∆F-BMP-FA, the resulting complex (BMP-A-Ab) is highly suitable for magnetic separation techniques and immunomagnetic assays. In comparison with commercial immunomagnetic beads, BMP-A-Ab had much higher capacity for adsorption of Vp (antigen) and gentamicin (hapten). The amount of BMP-A-Ab (in comparison with commercial immunomagnetic beads) required for such applications is low.

Commercial immunomagnetic beads are widely used in research and clinical labs, but are expensive (> $3 for 1 mg MB-A). Such expense presents a major obstacle to high-throughput screening in various routine detection and diagnostic assays (e.g., for pathogens or pollutants). In striking contrast, taking into account costs of electricity and culture media (but not labor), 1 mg ∆F-BMP-FA costs only $0.067, and is thus more feasible for high-throughput screening.

## Conclusion

Engineered BMPs are biogenic magnetic nanomaterials well-known for their eco-friendly properties. Because of the strict, difficult culture conditions required for recombinant bacterial strains, engineered BMPs are often considered unsuitable for practical applications. We developed a new engineered BMP fused with protein A (∆F-BMP-FA), demonstrated that its complex with Ab (BMP-A-Ab) has high capacity for adsorption of Vp (antigen) and gentamicin (hapten), and compared properties and costs of ∆F-BMP-FA with those of commercial immunomagnetic beads. Our findings indicate that engineered BMPs such as ∆F-BMP-FA are inexpensive, eco-friendly synthetic nanoparticles with strong potential applicability as alternatives to commercial immunomagnetic beads.

## Additional file

**Additional file 1.** Standard curves of Spa (protein A) and anti-gentamicin. **Figure S1.** Spa (protein A) standard curve determined by one-step ELISA. **Figure S2.** Standard curve of anti-gentamicin Ab determined by one-step ELISA.

## Abbreviations

BMP: magnetosome
Abs: antibodies
Vp: *Vibrio parahaemolyticus*
∆F-BMP-FA: recombinant magnetosome display proteinA on it
MB-A: commercial immunomagnetic beads
BMP-A-Ab: antibody and ∆F-BMP-FA complex
MB-A-Ab: antibody and MB-A complex
