# Supplementary material for: Engineered magnetosomes fused to functional molecule (protein A) provide a highly effective alternative to commercial immunomagnetic beads
Source: J Nanobiotechnology. 2019 Mar 6;17:37. doi: 10.1186/s12951-019-0469-z (PMC6402170; doi:10.1186/s12951-019-0469-z)
Supplement: Supplementary file 1 — Additional file 1. Standard curves of Spa (protein A) and anti-gentamicin. Figure S1. Spa (protein A) standard curve determined by one-step ELISA. Figure S2. Standard curve of anti-gentamicin Ab determined by one-step ELISA. [file 12951_2019_469_MOESM1_ESM.docx]

Additional file 1 Standard Curves of Spa (protein A) and Anti-Gentamicin Antibodies


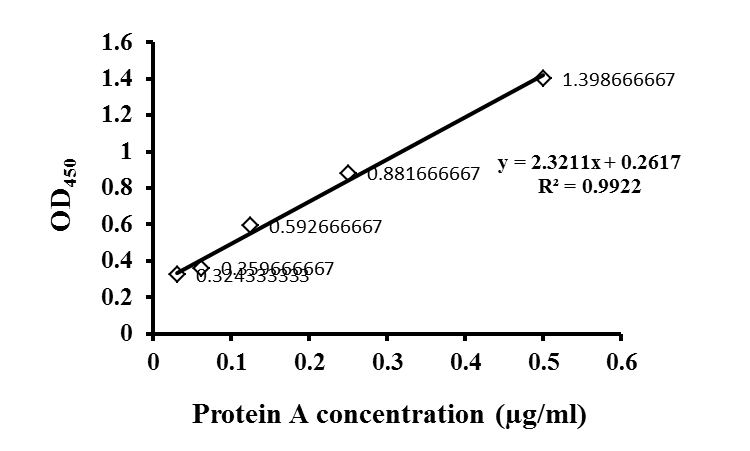


**Figure S1.** Spa (protein A) standard curve determined by one-step ELISA.


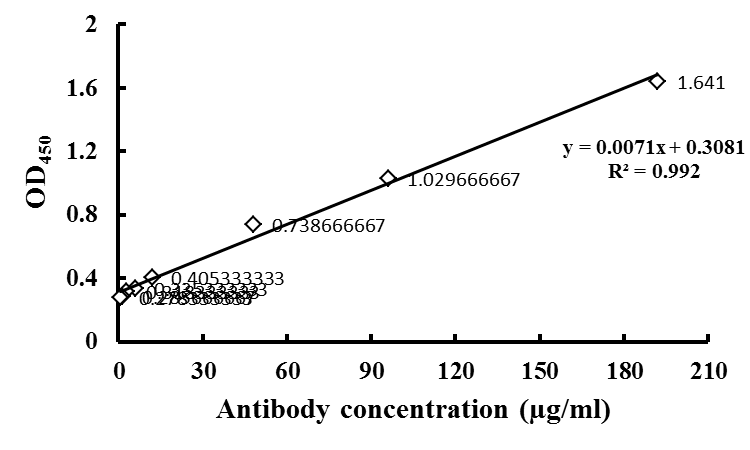


**Figure S2.** Standard curve of anti-gentamicin Ab determined by one-step ELISA.
